# The Role of Personality, Political Attitudes and Socio-Demographic Characteristics in Explaining Individual Differences in Fear of Coronavirus: A Comparison Over Time and Across Countries

**DOI:** 10.3389/fpsyg.2020.552305

**Published:** 2020-09-18

**Authors:** Julia V. Lippold, Julia I. Laske, Svea A. Hogeterp, Éilish Duke, Thomas Grünhage, Martin Reuter

**Affiliations:** ^1^Department of Psychology, University of Bonn, Bonn, Germany; ^2^Department of Psychology, University of Huddersfield, Huddersfield, United Kingdom; ^3^Laboratory of Neurogenetics, Center for Economics and Neuroscience, University of Bonn, Bonn, Germany

**Keywords:** Coronavirus, fear, politics, individual differences, cross-cultural, time course, personality, neuroticism

## Abstract

Since the emergence of the SARS-CoV-2 pandemic in December 2019 about 500,000 people died within the first 6 months. The virus itself, as well as the related political decisions, intensified an increasing feeling of fear in billions of people worldwide. However, while some people remained unperturbed, others experienced panic over the current situation. In order to investigate individual differences in the perceptions, emotions and behaviors in response to the Coronavirus pandemic, an online survey was conducted between 6th and 27th of March 2020. Participants included 7309 individuals from 96 countries, who provided information on socio-demographics, personality, political orientation and general life satisfaction. To determine the specificity of fear of Coronavirus, we also investigated fear related to two other current political issues: the refugee and the climate crises. Overall, in parallel with the escalation of the pandemic, fear of Coronavirus increased significantly over the 22-day period, with the strongest predictors being the personality variable neuroticism, as well as education, sex and being an at-risk person. A detailed longitudinal analysis of the largest sample, Germany, revealed that political orientation was also an important predictor of fear of Coronavirus. Specifically, conservatives were more afraid of Coronavirus than liberals. However, as the perceived threat of the virus increased, the influence of political orientation disappeared, whereas personality remained a stable predictor. The pattern of results regarding the perceived threat of the refugee and climate crises painted a different picture: political orientation was by far the best predictor, more important even than personality. Conservatives were more worried about the refugees, and liberals about climate change. Cross-cultural analyses showed pronounced differences between countries, dependent on the crisis. Nonetheless, the importance of personality for the prediction of fear of Coronavirus remained stable over time and across the world within the investigated 22-day period.

## Introduction

The Coronavirus (also known as SARS-CoV-2) causes the respiratory disease Covid-19 and represents the greatest health threat faced by mankind in decades, causing a steep increase in worldwide morbidity and mortality and eliciting widespread fear. Since its emergence in December 2019 in Wuhan, China, the virus spread rapidly around the globe and costed hundreds of thousands of lives within the first 6 months. The public health systems in some countries were on the verge of collapse, while the infection rate continued to rise. It was increasingly apparent that the threat of Coronavirus had been underestimated. Initially, the prevailing opinion was that the risk of Coronavirus is comparable to that of the common influenza virus. As the public realized the implications of the lack of available treatments for Covid-19, and that younger people without any pre-existing conditions can die from the disease, associated levels of fear and the perceived threat of the Coronavirus subjectively increased. The consequences of this were manifold. To curb the rapid infection rate, many governments took strong measures, such as shutting down wide areas and imposing restrictions on movement and freedom of assembly. For most people, social life had been reduced to a minimum. Shops were closed, companies halted production and services, millions of people were working from home, had been reduced to part-time work or lost their jobs. The media reported widespread instances of “panic buying,” meaning people were purchasing and hoarding groceries in vast quantities, particularly toilet paper, soap, disinfectant, and food. Many people were afraid of leaving the house. The pandemic also highlighted aspects of moral behavior, e.g., many people had volunteered to help others, either directly in the healthcare system or in their neighborhoods, by supporting elderly and at-risk neighbors. In contrast, some people continued to deny any potential danger and disobeyed political restrictions. The Corona crisis revealed all extremes of human behavior from panic to irresponsible ignorance, and from egoism to selfless altruism. What are the reasons for this variation in human behavior? Is the fear of Coronavirus specific, or are the people who panic in response to the virus also afraid of other perceived societal threats?

To address these questions, we launched an online survey, assessing personality and perceptions of social threat. This survey was initially only available in Germany, however, after 1 week, we created an English language version, which was available internationally. From a theoretical perspective, personality variables are the most promising starting point to address questions about individual differences in behavior, because personality is defined as the predisposition to respond to a certain class of stimuli with a certain class of behaviors, and these stimulus-response configurations are stable over time (Montag and Reuter, 2014). Therefore, it can be assumed that people with high scores on personality scales related to fear or anxiety are more prone to react with panic to the Corona crisis. Clinical research indicates the existence of specific phobias, e.g., arachnophobia, agoraphobia, claustrophobia etc. This means that people can be quite fearless in general, but have an extreme fear of a specific object or situation. To control for this, we considered participants’ levels of fear of other current political crises – climate change and refugees – in addition to their fear of Coronavirus. Political orientation is a key predictor of attitudes to climate change and the refugee crisis. Green political parties are concerned with climate change, right-wing political parties tend to argue against the inclusion of refugees, and most importantly for the present study, also tend to be fearful of contaminants and infections, which may explain their nationalistic and xenophobic stances (Schaller et al., 2015).

Fear and anxiety belong to the basic set of emotions common to all ethnicities and cultures and to non-human mammals (Ekman, 2006). Consequently, fear and anxiety have strong evolutionary relevance, signaling threat and danger and therefore protecting the individual and promoting survival (Reuter et al., 2015). However, extreme forms of fear and anxiety are not adaptive; they prevent people from being satisfied with life and being a functional member of society (Lahey, 2009). Similar to other personality dimensions, fear and anxiety are normally distributed in the population, i.e., most people have medium levels, while relatively few people have extremely low or high levels of fear or anxiety. This frequency distribution provides us with meaningful information on the reasons for individual differences in these emotional systems, i.e., many independent factors must interact to create a normal distribution (Gangestad and Snyder, 1985). From twin studies, we know that genetic and environmental factors account for about 50% of the variance in personality (Plomin and Asbury, 2005). Therefore, many genes and environmental factors work together to shape an individual’s personality. In extreme situations, like the present Coronavirus pandemic, it is likely that the situational factors become more dominant, reducing the influence of the personality traits.

All personality theories have at least one dimension representing the predisposition of sensitivity to negative stimuli, and thus a vulnerability for anxiety disorders. Neuroticism is arguably the best-known example of such traits. Neurotic individuals are anxious, moody, tense, tend to worry and are often depressed (Caspi et al., 2005). Neuroticism is one of the five traits described by the Big-5 personality theory (Costa and McCrae, 1992). In the neurosciences, more biologically oriented personality theories are preferred, e.g., Jeffrey Gray’s reinforcement sensitivity theory (RST), which, in its revised form, differentiates between fear and anxiety (Gray and McNaughton, 2000). One of the strongest arguments for this differentiation between fear and anxiety is that only the latter can be influenced by pharmaceutical drugs (i.e., anxiolytic drugs like benzodiazepines), although there is some overlap in the neuroanatomical circuits underpinning the two constructs (McNaughton and Gray, 2000; Lippold et al., 2020). The main differences between these two concepts is that fear represents negative situations we absolutely want to avoid, whereas anxiety is related to negative situations we nonetheless want to approach (e.g., an exam; if we do not engage with the exam, we cannot pass it). However, it is evident that few, if any, individuals will show approach behavior to the virus (i.e., anxiety-related behavior), notable exceptions here may be scientists researching possible treatments, and people from the healthcare system supporting patients. From this perspective the pandemic is predominantly causing avoidance behavior and, therefore, it is a situation that should evoke fear rather than anxiety. However, anxiety is also triggered in situations where an individual is confronted with a new and, therefore, unpredictable stimulus (Gray and McNaughton, 2000). Coronavirus is new and unknown to us; its consequences not yet predictable. Therefore, behavior related to both anxiety and fear are plausible reactions to the crisis. Thus, associations between fear related to Coronavirus and self-reported negative personality traits will provide excellent validation data for measurement tools assessing either fear or anxiety.

In addition to personality and political attitudes, socio-demographic variables are of interest. Are there gender- or age- related differences in fear of Coronavirus, or does education level influence how people cope with the pandemic? It is well established that women tend to be more anxious in general, relative to men (Toufexis et al., 2006), but is this also true for the fear of a virus? The mass media bombards us with ever-changing information about Coronavirus, and recipients must filter this information and decide which sources are trustworthy and which merely offer clickbait or fake news. While the capacity to effectively filter information is related to an individual’s level of education (Peters et al., 2018), this is not the only factor: A selection bias in the perception of stimuli is a well-established endophenotype of neuroticism and related affective disorders (Mogg et al., 1993). Neurotic individuals and patients with anxiety disorders tend to selectively filter negative information. Thus, higher levels of neuroticism may be one explanation for why some people have greater fear of Coronavirus.

The aim of our study is to explain individual differences in the fear of Coronavirus, considering both changes in fear levels over time (in a between-subjects design) and comparisons between different countries. In addition, we want to investigate the specificity of fear of Coronavirus by comparing it with two other current political issues; the refugee and the climate crises.

## Materials and Methods

### Study Design/Study Protocol

An online study was run between 6th and 27th of March 2020, aiming to identify personality traits and socio-demographic predictors of individual differences in fear of Coronavirus. During the first 6 days, a longer (25 min) version of the study was run, restricted to a German speaking sample. We subsequently translated the study to English, shortened it to 15 min, and made it available internationally. We amended the original German survey to correspond with this new English-language version. The reason for shortening the survey was to increase participation. The original version contained a longer, more nuanced personality measure. Therefore, analyses of the cross-cultural data started on day 7, when the international survey was launched. Participant recruitment was carried out via social media, such as Twitter and Facebook. Participation was completely anonymous and was not incentivized. Participants provided informed consent before beginning the study, which was conducted in accordance with the ethical declaration of Helsinki (World Health Organization [WHO], 2001). Upon completion of the study, each participant received individualized feedback on their personality, based on the answers given to the revised Reinforcement Sensitivity Theory Questionnaire (r-RST-Q) (Reuter et al., 2015).

### Sample

In total, 7309 participants from 96 different countries completed the survey (*M*_age_ = 33.23, SD = 11.78, range: 18–89; females = 5611, males = 1661, other = 37). The cross-cultural analyses included the following 13 countries and group of countries: Germany (*N* = 3469), Denmark (*N* = 662), Great Britain (*N* = 387), Eastern Europe (*N* = 332; including Bulgaria, Czechia, Hungary, Lithuania, Moldova, Poland, Romania, Russia, Ukraine, Slovakia), United States (*N* = 282), Netherlands (*N* = 251), Italy (*N* = 225), former Yugoslavia (*N* = 197; including Bosnia and Herzegovina, Croatia, Kosovo, Macedonia, Montenegro, Serbia, Slovenia), France (*N* = 192), Ireland (*N* = 158), Australia and New Zealand (*N* = 164), Austria (*N* = 118), and Sweden (*N* = 94). The grouping of nations was based on cultural, geographical and historical similarities.

A detailed overview of the characteristics of the respective countries can be found in the Supplementary Table 1. Other countries where the sample sizes were too small to permit individualized evaluation were only considered in the overall analyses, i.e., as part of the total international sample.

### Measures

Socio-demographic information, including age, gender and educational level were obtained. Participants were also asked to indicate whether they were at heightened risk of Coronavirus due to age (>60 years) or pre-existing illness. Furthermore, we included questions regarding general life satisfaction (six-point Likert scale) and political orientation (seven-point Likert scale, ranging from left to right). Personality was assessed using the 10-item short version of the Big Five Inventory (BFI) (Rammstedt and John, 2007). The behavioral activation system (BAS), behavioral inhibition system (BIS) (a measure of anxiety), and the Fight-Flight-Freeze System (FFFS) (reflecting fear) were measured using the r-RST-Q (Reuter et al., 2015). Descriptive statistics for the questionnaires (BFI and r-RST-Q) are available in the Supplementary Table 2 for the countries and country groups described above. Participants indicated their level of fear regarding each of the following: Coronavirus (“To what degree are you worried about COVID-19?,” using a six-point Likert scale), climate change (“To what extent do you experience feelings of anxiety and threat because of the climate crisis? Because of general discomfort about the climate crisis,” using a four-point Likert scale) and the refugee crisis (“To what extent do you experience feelings of anxiety and threat because of the refugee crisis? Please indicate the extent to which you agree/disagree with each of the following statements. Because I have a feeling of general discomfort, using a four-point Likert scale). This latter category was only shown to participants who indicated that they live in a country that hosts or acts as a transit for refugees. Descriptive statistics for these three dependent variables are also given in the Supplementary Table 3. For the cross-cultural comparison, only data collected from day 7 onward were considered (to prevent bias in the German sample, where data had been collected prior to the worsening of the crisis).

### Statistical Analyses

To test the effects of the independent variables time (days of study) and sex on the dependent variable “fear of Coronavirus,” we conducted a two-factorial analysis of variance (ANOVA). Multivariate linear regression models were calculated to identify the best predictors for the dependent variables (fear of: Coronavirus; refugees; and climate change). Predictors [i.e., personality: rRST-Q variables (BIS, BAS, FFFS), BFI-Big-5 variables (extraversion, agreeableness, neuroticism, openness, conscientiousness), life satisfaction, political orientation, sex, age, education, being an at risk person] were entered stepwise (inclusion criteria: *F*-value probability < 0.05, exclusion criteria: *F* > 0.10) to obtain the most parsimonious model. Pairwise deletion of variables was applied. In the interpretation of results, only predictors explaining ≥5% of incremental variance were considered. In addition, Pearson correlations were calculated to test for associations between political orientation and the three perceived threats (i.e., Coronavirus, refugee crisis, climate change). In the cross-cultural analyses, ANCOVA models were calculated for each of the three fears, using each country/group of countries as a between-subjects factor and age and level of education as covariates. For *post hoc* comparisons between groups (countries) simple contrasts were calculated.

## Results

### Increase in Fear of Coronavirus Over Time (22-Day Interval) in the Total Sample

**FIGURE 1:**
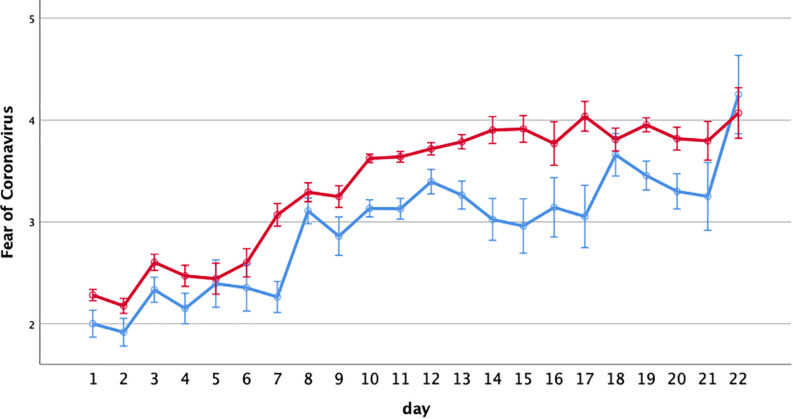
Increase in fear of Coronavirus over time (22-day interval; cross-sectional design, total sample) dependent on gender (blue line: men; red line: women). The data collection ran between March 6th and 27th 2020. Depicted are means ±1 SEM.

Results for the total sample showed significant main effects of time (i.e., escalating fear throughout the 22-day study period) (*F*_(21,7228)_ = 41.61, *p* ≤ 0.00001, eta^2^ = 0.108) and sex (*F*_(1,7228)_ = 71.36, *p* ≤ 0.00001, eta^2^ = 0.010). Perceived fear of Coronavirus increased over time and was significantly higher among women compared to men (see Figure 1).

### Predictors of Fear of Coronavirus in the Total Sample

Multiple linear regression analysis revealed that the four variables neuroticism (β = 0.197), education (β = 0.164), sex (β = 0.085), and being an at-risk person (β = 0.079) could significantly explain variance in fear of Coronavirus (*R*^2^ = 0.082; *F*_(4,6308)_ = 141.62, *p* ≤ 0.00001). High neuroticism, having a higher level of education, being female, and being an at-risk person (e.g., due to age or health) emerged as the strongest predictors of higher levels of fear of Coronavirus.

### Predictors of Fear of Coronavirus in the German Sample

The German sample also revealed an increase in fear of Coronavirus over time (*F*_(21,3411)_ = 14.69, *p* ≤ 0.00001, eta^2^ = 0.083) and an influence of sex, with women being more afraid than men (*F*_(1,3411)_ = 25.75, *p* ≤ 0.00001, eta^2^ = 0.007). Confining the analysis to the German sample (*R*^2^ = 0.059; *F*_(3,2__941__)_ = 62.77, *p* ≤ 0.00001), two predictors significant for the total sample, neuroticism (β = 0.230) and education (β = 0.076), were replicated. However, the predictive ability of sex and being an at-risk person was replaced by political orientation (β = 0.075) for this sample. Right-leaning political orientation was associated with greater fear of Coronavirus. Although sex did not emerge as a significant predictor for the German sample, significantly higher levels of fear were observed for women relative to men (see above).

### Predictors of Fear of Coronavirus Dependant on Time in the German Sample

Due to the fact that data collection in the German sample 415 started 6 days before the international survey was launched, we could consider the effect of time on the predictors of our dependent variable. As the most pronounced increase in fear of Coronavirus was observed between days 7 and 8, we recalculated the regression models in two different time periods: days 1–7 (period I) and days 8–22 (period II). For period I, the model (*R*^2^ = 0.073; *F*_(3,1665)_ = 44.73, *p* ≤ 0.00001) revealed three predictors; neuroticism (β = 0.234), political orientation (β = 0.113), and being considered high-risk (β = 0.094). Higher neuroticism, right-ward political orientation, and belonging to the at-risk group predicted greater fear of Coronavirus during the initial days of the study.

Three predictors emerged for period II (*R*^2^ = 0.087; *F*_(3,1268)_ = 41.45, *p* ≤ 0.00001); neuroticism (β = 0.247), education (β = 0.105), and sex (β = 0.088). Besides higher neuroticism, having a higher level of education and being female were the best predictors of heightened fear during the later period of the study.

### Predictors of Fear of Refugees in the Total Sample

A different picture emerged for results pertaining to perceived threat from the refugee crisis. The multiple regression model for the total sample (*R*^2^ = 0.150; *F*_(6,4465)_ = 132.04, *p* ≤ 0.00001) showed that political orientation (β = 0.317) was the strongest predictor, followed by education (β = −0.108), life satisfaction (β = −0.070), age (β = 0.107), agreeableness (β = −0.079), and anxiety (BIS; β = 0.071). People with a more conservative political orientation, a lower level of education, lower general life satisfaction, of older age, low agreeableness and high anxiety have higher levels of fear about the threat posed by refugees.

### Predictors of Fear of Climate Change in the Total Sample

The climate change model explained substantially less variance than did the fear of refugees model (*R*^2^ = 0.086; *F*_(5,6307)_ = 104.59, *p* ≤ 0.00001). However, political orientation again emerged as the strongest predictor (β = −0.169), followed by anxiety (BIS; β = 0.094), sex (β = 0.092), education (β = 0.092), age (β = −0.071), and neuroticism (β = 0.069). For this model, a liberal political orientation, high anxiety and neuroticism, high education, being female and young are associated with greater fear of climate change.

### Results of the Cross-Cultural Data

**FIGURE 2:**
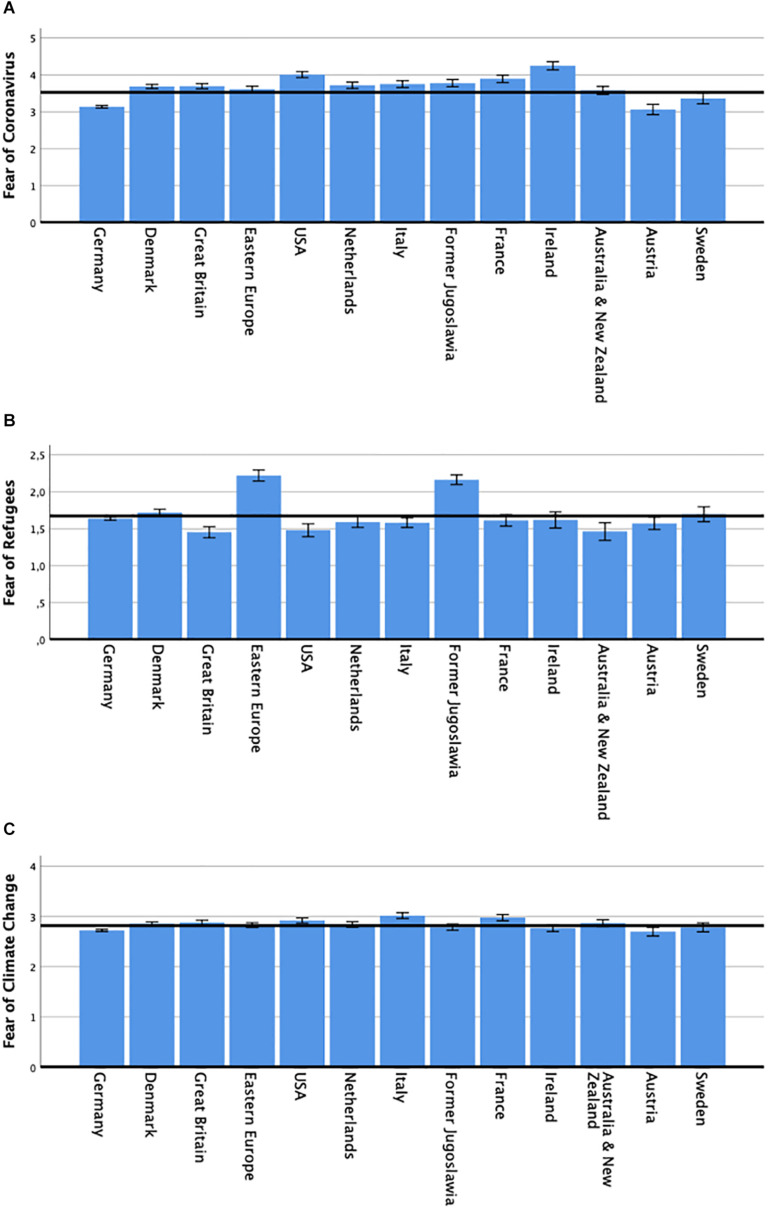
Cross-cultural comparisons **(A)** Levels of fear of Coronavirus per country (*n* = 6531). **(B)** Fear of perceived threat of refugees per country (*n* = 4831). **(C)** Fear of climate change per country (*n* = 6531). The horizontal line in each graph marks the average score for the total sample. Depicted are means ±1 SEM.

Results of the cross-cultural data (starting on day 7, when the international survey was launched) showed significant differences between countries (*F*_(12,4552)_ = 21.26, *p* ≤ 0.00001, eta^2^ = 0.053). As can be seen from Figure 2A, fear of the Coronavirus was lowest in Germany, Austria, and Sweden, where the mean fear scores were markedly below the average for the total sample, and differed significantly from those of other countries, as indicated by *post hoc* contrasts. The country reporting the greatest fear of Coronavirus, Ireland, had significantly higher scores in comparison to all other countries, except the United States.

With respect to fear of the threat posed by refugees, there were also significant differences between countries (*F*_(12,2852)_ = 11.12, *p* ≤ 0.00001, eta^2^ = 0.045). States of Eastern Europe and states of the former Yugoslavia reported fear scores that were significantly higher than in all other countries (see Figure 2B). Significant between-countries differences were also observed for fear of climate change (*F*_(12,4552)_ = 3.94, *p* ≤ 0.00001, eta^2^ = 0.010). France, Italy, and the United States reported higher than average fear, while those of Germany and Austria were below average (see Figure 2C).

## Discussion

In a cross-cultural study covering a 22-day period, during which the Coronavirus pandemic escalated worldwide, we investigated why some people are more afraid of the Coronavirus than others. Our results show a marked increase in the fear of Coronavirus over time in a German sample, a trend echoed in a wider, international sample. For all countries, women reported significantly higher levels of fear of Coronavirus than men. This increase in fear over time reflects the growing infection rates and the increasingly severe governmental decisions and sanctions aimed at fighting the pandemic in March 2020. In both the international and the German samples, fear of the Coronavirus was best predicted by personality. The personality trait neuroticism – assessed via the short BFI (Rammstedt and John, 2007) – emerged as the strongest predictor, and its ability to explain the perceived threat of Coronavirus seemed to remain relatively stable, over time and country. In the international sample, education, gender and being an at-risk person also predicted fear level. Being more neurotic, female, politically conservative and having a higher level of education, are all factors related to fear of Coronavirus. However, when the analysis was restricted to the large German sample, political orientation proved important and replaced the latter two predictors.

The key predictor of the level of perceived threat of Coronavirus was neuroticism. Originally proposed as one of the key personality dimensions by Eysenck (1991), neuroticism also forms part of the Big-5 personality theory, the reliability and validity of which has been documented in countless cultures around the globe (Costa and McCrae, 1992). Although originally derived from the former psychoanalytic diagnosis “neurosis,” neuroticism has nothing to do with mental illness. It describes the propensity to be shy, anxious, moody, easily depressed, vulnerable, and self-conscious. Moreover, high-N people are more likely to report health problems and tend to exaggerate concerns about their state of health (Innes and Kitto, 1989). While neuroticism varies throughout the healthy population, high neuroticism is a well-established risk factor for numerous psychopathologies and psychosomatic complaints (Lahey, 2009). There is also evidence from molecular genetics that neuroticism and mental illnesses share the same candidate genes (Canli and Lesch, 2007). However, neuroticism is clearly a mixture of both anxiety and fear, demonstrating highly significant correlations with fear (RST-FFFS) as well as with anxiety (RST-BIS) in the present sample (*r* = 0.438 and *r* = 0.479, respectively). Given the overlap in neural circuitry and neurochemistry between fear and anxiety, the strong intercorrelation between these constructs (*r* = 0.596) is unsurprising (Gray and McNaughton, 2000). Clearly, neuroticism accounts for the shared variance between fear and anxiety, because the later personality traits did not account for additional variance in fear of Coronavirus, beyond that explained by neuroticism. This thesis is corroborated by imaging studies reporting higher amygdala responsivity to negative stimuli under stress in neurotic individuals (Everaerd et al., 2015) and a positive association between the concentration of gray matter in the amygdala and neuroticism (Omura et al., 2005). The amygdala is doubtless the core region for the processing of fear and anxiety in the mammalian brain (Gray and McNaughton, 2000; LeDoux, 2003; Panksepp, 2011). Thus, the role of neuroticism in predicting fear of Coronavirus is likely to be driven by evolutionary factors. Fear and anxiety are among the oldest emotions, originating from the limbic system, which includes the amygdala (Panksepp, 2011). In line with this, fear of contamination and infection is highly adaptive for survival (e.g., Schaller et al., 2015).

Data for the full 22-day period were only available for the German sample. A peak in the increase of fear of Coronavirus was observed between day 7 and 8 (i.e., March 12th to 13th). March 13th marked the closure of all schools and kindergartens in Germany, which made the severity of the situation much more evident to the general public. When the two time periods, i.e., pre- and post-school closures, are contrasted, the personality dimension of neuroticism remained important in the German sample. However, the influence of other predictors, e.g., political orientation and being an at-risk person appeared to be transient. Political orientation, in particular, seemed to be less important the more severe the crisis became. During the early stages of the pandemic when infection rates were low, people who identified with more conservative ideologies reported greater fear of the virus than did voters who favor more liberal parties. This supports well-documented findings from the literature of higher disgust sensitivity and fear of contamination and infection among conservatives (e.g., Inbar et al., 2009). In line with this, Navarrete and Fessler (2006) found that individuals who perceived themselves at greater risk for infectious disease expressed more ethnocentric attitudes and Thornhill et al. (2009) even suggested that pathogen threats not only motivate intergroup bias and ethnocentrism, but also promote a conservative political ideology.

Political orientation was also of interest with regards to the refugee and climate crises, since it was the strongest predictor of both issues. Data from the German sample suggested an association between a more conservative political orientation and greater perceived threat from refugees (period I: *r* = 0.366; period II: *r* = 0.333), as well as less fear over climate change (period I: *r* = −0.211; period II: *r* = −0.226). These results were consistent across both time intervals. However, the associations between political orientation and fear of the Coronavirus (period I: *r* = 0.107; period II: *r* = 0.003) was only apparent during period I.

The role of political orientation as a predictor of perceived threat was particularly interesting in the present study. Previous research has established links between conservatism and negative attitudes toward refugees (Anderson and Ferguson, 2018) as well as less concern about climate change (McCright and Dunlap, 2011), findings that were echoed in the present study. However, while these findings remained stable over the two time periods of the current study, the associations between political orientation and fear of the Coronavirus showed a different pattern. Specifically, as the crisis intensified, political orientation ceased to predict fear of Coronavirus. Similar findings were observed for the international sample, whereby conservatism was related to fear of refugees (*r* = 0.309), while liberalism was linked to fear of climate change (*r* = −0.201). In the total sample, political orientation was not associated with fear of Coronavirus. Two limitations should be noted in this respect; first, data are not available for days 1–6 for the international sample, i.e., before the intensification of the crisis, e.g., through closing of schools etc.; second, our study did not set out to explicitly examine changes in political orientation, thus no causal conclusions can be made. It seems logical to assume, however, that the influence of political orientation on fear of climate change, fear of refugees or any other potential crisis would also disappear, once the public perception of these crises became life threatening. In this respect, the present findings raise important implications for the messaging around climate change and refugee aid.

The present results also highlighted differences in how each of the three threats were perceived internationally. Eastern European states and states belonging to former Yugoslavia were more afraid of refugees; Italians and the French were more worried about climate change. Interestingly, in Germany, Austria and Sweden, the perceived threat of Coronavirus was lowest. One possible explanation for this finding is that these countries have good healthcare systems and that Coronavirus lethality rates were quite low there compared to other countries over the investigated 22-day period. Most importantly, the comparison of 767 the predictors of the three threats showed that the personality 768 trait neuroticism best predicted fear of Coronavirus, whereas 769 political orientation played a dominant role in predicting fear of 770 refugees and climate change.

Lower levels of education predicted greater fear of refugees in the total sample. The mass media presents us with infinite information on political crises, encompassing both evidence-based facts and recommendations, as well as many “fake news” stories, e.g., that the climate change is a great swindle. A higher level of education helps people to filter information and to prevent panic (Peters et al., 2018). In the current study, this finding was augmented by small, but nonetheless significant, results indicating that people with a higher educational level believed they can inform themselves more objectively (*r* = 0.102) and did not believe that information was being deliberately withheld by authorities (*r* = −0.068). Our data also indicates that the perceived objectivity of media reportage is associated with fear of the virus. People who believed that the media downplays the severity of the crisis (*r* = 0.288) or deliberately withheld information (*r* = 0.219) were more afraid.

It has to be pointed out that the present study has some limitations that deserve discussion. First, cross-cultural studies have always the problem that not all country-specific differences can be controlled for. However, in the present study the cross-cultural data included only days 7–22 and day 7 is March 12th, the day after the WHO has declared the Coronavirus a global pandemic. This means that despite differences in infection rates across countries, the virus was present as a threat in the population worldwide. There are also differences in the salience of the refugee crisis across countries. But this salience is largely dependent on the subjective perception rather on the objective threat. For example, in Europe the number of refugees in a country is not correlated with the extent of xenophobia. In order to minimize a possible bias, we excluded participants who stated that their country is not a target or transit country for refugees. Another methodological shortcoming refers to the single item measure of the three dependent variables (fear of Coronavirus, fear of refugees, fear of climate change). Although multiple item scales are preferable (only for these reliability measures can be calculated) we refrained from adding additional items because we did not find that other items capture additional aspects that were not at least implicitly included in our global item.

## Conclusion

In conclusion, this study aimed to provide insight into how personality, demographic factors (age, sex, education) and political attitudes influence the perception of threat caused by the Coronavirus. The data indicate that the personality variable neuroticism, related to negative emotionality, predicted higher perceived threat from Coronavirus. Neuroticism outweighed the contribution of other important factors, including political orientation, gender and education level. These data raise practical points, which governments need to consider to decrease the public’s fear of Coronavirus, including a push for clear messaging around the virus, stronger quality control among media outlets to promote objectivity and reduce the prevalence of “fake news” stories, and increased promotion of – and support for – mental health organizations, which have a valuable role to play in helping the public to manage anxiety at this time.

## Data Availability Statement

The dataset presented in the study can be found at https://osf.io/b5yxc/?view_only=6ad94a8fed194bda845f29778a27ab03.

## Ethics Statement

Ethical review and approval was not required for the study on human participants in accordance with the local legislation and institutional requirements. The patients/participants provided their written informed consent to participate in this study.

## Author Contributions

JVL: conceptualization, project administration, programming, writing, statistical analyses, and data curation. JIL: conceptualization, programming, and data curation. SH: programming and data curation. ÉD: review and editing and consulting. TG: conceptualization and review and editing. MR: conceptualization, supervision, writing, and statistical analyses. All authors contributed to the article and approved the submitted version.

## Conflict of Interest

The authors declare that the research was conducted in the absence of any commercial or financial relationships that could be construed as a potential conflict of interest.
