# Supplementary material for: The Role of Personality, Political Attitudes and Socio-Demographic Characteristics in Explaining Individual Differences in Fear of Coronavirus: A Comparison Over Time and Across Countries
Source: Front Psychol. 2020 Sep 18;11:552305. doi: 10.3389/fpsyg.2020.552305 (PMC7530433; doi:10.3389/fpsyg.2020.552305)
Supplement: Supplementary file 1 [file Table_1.DOCX]

Supplementary Material


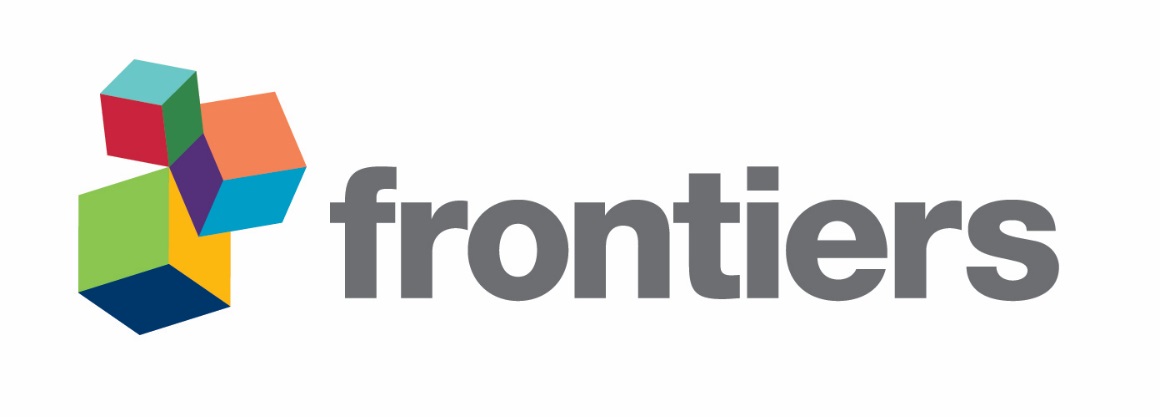


Table S1.

Tab. S1: Descriptive statistics for countries and group of countries.

| *Country* | *N* | *sex* | | | *age* | | |
| --- | --- | --- | --- | --- | --- | --- | --- |
|  |  | ♂ | ♀ | ⚧ | *M* | *SD* | *range* |
| Total Sample | 7309 | 1661  (22.7 %) | 5611  (76.8 %) | 37  (0.5 %) | 33.23 | 11.78 | 18-89 |
| Germany  (all) | 3469 | 884  (25.5 %) | 2571  (74.1 %) | 14  (0.4 %) | 32.89 | 12.29 | 18-89 |
| Germany  (day 7 - 22) | 1553 | 450  (29.0 %) | 1097  (70.6 %) | 6  (0.4 %) | 32.12 | 11.93 | 18-88 |
| Denmark | 662 | 131  (19.8 %) | 530  (80.1 %) | 1  (0.2 %) | 29.72 | 7.79 | 18-67 |
| Great Britain | 387 | 65  (16.8 %) | 320  (82.7 %) | 2  (0.5 %) | 37.95 | 12.49 | 18-73 |
| Eastern Europe | 332 | 68  (20.5 %) | 263  (79.2 %) | 1  (0.3 %) | 31.97 | 8.91 | 19-67 |
| USA | 282 | 41  (14.5 %) | 236  (83.7 %) | 5  (1.8 %) | 40.09 | 14.36 | 18-80 |
| Netherlands | 251 | 49  (19.5 %) | 200  (79.7 %) | 2  (0.8 %) | 30.75 | 9.24 | 18-72 |
| Italy | 225 | 56  (24.9 %) | 169  (75.1 %) | - | 32.74 | 10.89 | 18-75 |
| Former Yugoslavia | 197 | 24  (12.2 %) | 173  (87.8 %) | - | 26.74 | 5.13 | 20-53 |
| France | 192 | 42  (21.9 %) | 148  (77.1 %) | 2  (1.0 %) | 35.47 | 13.57 | 18-76 |
| Ireland | 158 | 25  (15.8 %) | 132  (83.5 %) | 1  (0.6 %) | 37.90 | 11.43 | 18-77 |
| Australia  & New Zealand | 164 | 21  (12.8 %) | 138  (84.1 %) | 5  (3.0 %) | 39.41 | 11.87 | 20-77 |
| Austria  (all) | 118 | 35  (29.7 %) | 83  (70.3 %) | - | 33.15 | 9.97 | 18-69 |
| Austria  (day 7 - 22) | 96 | 24  (25.0 %) | 72  (75.0 %) | - | 33.34 | 9.50 | 19-69 |
| Sweden | 94 | 23  (24.5 %) | 69  (73.4 %) | 2  (2.1 %) | 32.45 | 10.46 | 20-72 |

Table S2.

Tab. S2. Descriptive statistics and reliabilities for questionnaires. Correlation coefficients refer to the BFI scales (Rammstedt & John, 2007); Cronbach’s α’s refer to the rRST-Q scales (Reuter et al., 2015).

| *Country* | *Questionnaire* | *N* | *Scale* | *M* | *SD* | *α / r* |
| --- | --- | --- | --- | --- | --- | --- |
| Total sample | BFI | 7067 | Neuroticism | 2.92 | 1.03 | .681 |
|  |  | 7042 | Agreeableness | 3.30 | .83 | .292 |
|  | rRST-Q | 6388 | FFFS | 2.33 | .41 | .774 |
|  |  | 6388 | BIS | 2.49 | .47 | .807 |
| Germany | BFI | 3353 | Neuroticism | 2.92 | .97 | .648 |
|  |  | 3350 | Agreeableness | 3.20 | .78 | .270 |
|  | rRST-Q | 2967 | FFFS | 2.33 | .43 | .793 |
|  |  | 2967 | BIS | 2.48 | .48 | .823 |
| Germany  (day 7 - 22) | BFI | 1525 | Neuroticism | 2.89 | .98 | .655 |
|  |  | 1525 | Agreeableness | 3.25 | .80 | .286 |
|  | rRST-Q | 1434 | FFFS | 2.34 | .42 | .784 |
|  |  | 1434 | BIS | 2.48 | .47 | .810 |
| Denmark | BFI | 640 | Neuroticism | 2.88 | 1.12 | .754 |
|  |  | 640 | Agreeableness | 3.39 | .87 | .296 |
|  | rRST-Q | 595 | FFFS | 2.35 | .39 | .766 |
|  |  | 595 | BIS | 2.53 | .47 | .808 |
| Great Britain | BFI | 374 | Neuroticism | 2.91 | 1.14 | .721 |
|  |  | 370 | Agreeableness | 3.36 | .87 | .291 |
|  | rRST-Q | 343 | FFFS | 2.35 | .42 | .794 |
|  |  | 343 | BIS | 2.54 | .45 | .798 |
| Eastern Europe | BFI | 315 | Neuroticism | 3.03 | 1.01 | .672 |
|  |  | 313 | Agreeableness | 3.49 | .83 | .209 |
|  | rRST-Q | 290 | FFFS | 2.35 | .34 | .705 |
|  |  | 290 | BIS | 2.46 | .42 | .782 |
| USA | BFI | 275 | Neuroticism | 3.01 | 1.10 | .690 |
|  |  | 275 | Agreeableness | 3.17 | .94 | .425 |
|  | rRST-Q | 253 | FFFS | 2.29 | .42 | .805 |
|  |  | 253 | BIS | 2.45 | .47 | .820 |
| Netherlands | BFI | 246 | Neuroticism | 2.92 | 1.10 | .726 |
|  |  | 245 | Agreeableness | 3.37 | .85 | .292 |
|  | rRST-Q | 227 | FFFS | 2.36 | .39 | .751 |
|  |  | 227 | BIS | 2.53 | .46 | .790 |
| Italy | BFI | 222 | Neuroticism | 2.77 | 1.06 | .691 |
|  |  | 218 | Agreeableness | 3.42 | .85 | .210 |
|  | rRST-Q | 199 | FFFS | 2.31 | .35 | .711 |
|  |  | 199 | BIS | 2.50 | .41 | .741 |
| Former Yugoslavia | BFI | 192 | Neuroticism | 2.97 | 1.12 | .749 |
|  |  | 191 | Agreeableness | 3.53 | .87 | .213 |
|  | rRST-Q | 179 | FFFS | 2.49 | .25 | .739 |
|  |  | 179 | BIS | 2.49 | .46 | .797 |
| France | BFI | 186 | Neuroticism | 3.04 | 1.06 | .719 |
|  |  | 186 | Agreeableness | 3.40 | .82 | .320 |
|  | rRST-Q | 172 | FFFS | 2.37 | .38 | .732 |
|  |  | 172 | BIS | 2.56 | .47 | .817 |
| Ireland | BFI | 149 | Neuroticism | 2.92 | 1.13 | .714 |
|  |  | 147 | Agreeableness | 3.29 | .87 | .225 |
|  | rRST-Q | 137 | FFFS | 2.34 | .43 | .787 |
|  |  | 137 | BIS | 2.53 | .48 | .839 |
| Australia  & New Zealand | BFI | 159 | Neuroticism | 3.00 | 1.09 | .693 |
|  |  | 156 | Agreeableness | 3.32 | .87 | .361 |
|  | rRST-Q | 144 | FFFS | 2.34 | .39 | .791 |
|  |  | 144 | BIS | 2.54 | .45 | .826 |
| Austria | BFI | 116 | Neuroticism | 2.77 | .98 | .642 |
|  |  | 116 | Agreeableness | 3.20 | .83 | .309 |
|  | rRST-Q | 110 | FFFS | 2.26 | .41 | .761 |
|  |  | 110 | BIS | 2.44 | .53 | .846 |
| Sweden | BFI | 93 | Neuroticism | 2.78 | 1.07 | .768 |
|  |  | 93 | Agreeableness | 3.57 | .90 | .482 |
|  | rRST-Q | 89 | FFFS | 2.32 | .41 | .785 |
|  |  | 89 | BIS | 2.51 | .47 | .791 |

**Table S3.**

Tab. S3: Descriptive statistics for dependent variables for all countries and group of countries.

| *Country* | *Fear of Coronavirus* | | *Fear of refugees* | | *Fear of climate change* | |
| --- | --- | --- | --- | --- | --- | --- |
|  | *M* | *SD* | *M* | *SD* | *M* | *SD* |
| Total Sample | 3.22 | 1.47 | 1.71 | .85 | 2.74 | .89 |
| Germany (all) | 2.67 | 1.34 | 1.70 | .84 | 2.62 | .92 |
| Germany (day 7 -22) | 3.12 | 1.38 | 1.66 | .80 | 2.72 | .92 |
| Denmark | 3.69 | 1.31 | 1.66 | .82 | 2.91 | .83 |
| Great Britain | 3.68 | 1.49 | 1.49 | .77 | 2.81 | .86 |
| Eastern Europe | 3.63 | 1.35 | 2.18 | .91 | 2.86 | .76 |
| USA | 4.01 | 1.39 | 1.55 | .77 | 2.84 | .85 |
| Netherlands | 3.70 | 1.34 | 1.57 | .80 | 2.88 | .81 |
| Italy | 3.77 | 1.35 | 1.54 | .74 | 3.04 | .80 |
| Former Yugoslavia | 3.79 | 1.19 | 2.08 | .98 | 2.87 | .79 |
| France | 3.90 | 1.36 | 1.61 | .79 | 2.95 | .95 |
| Ireland | 4.22 | 1.44 | 1.70 | .95 | 2.70 | .85 |
| Australia & New Zealand | 3.59 | 1.53 | 1.51 | .78 | 2.80 | .87 |
| Austria (all) | 2.90 | 1.30 | 1.58 | .80 | 2.59 | 1.02 |
| Austria (day 7 -22) | 3.05 | 1.21 | 1.58 | .80 | 2.69 | 0.99 |
| Sweden | 3.35 | 1.44 | 1.67 | .82 | 2.77 | .86 |
